# Multiple Cryptic Binding Sites are Necessary for Robust Fibronectin Assembly: An *In Silico* Study

**DOI:** 10.1038/s41598-017-18328-4

**Published:** 2017-12-22

**Authors:** Christopher A. Lemmon, Seth H. Weinberg

**Affiliations:** Department of Biomedical Engineering, Virginia Commonwealth University, Richmond, VA 23298 USA

## Abstract

The mechanism of assembly of the extracellular matrix protein fibronectin (FN) into elastic, insoluble fibrils is still poorly understood. FN fibrillogenesis requires cell-generated forces, which expose cryptic FN-FN binding sites buried in FN Type III domains. The number and location of cryptic binding sites have been debated, but experimental evidence suggests multiple domains may contain FN-FN binding sites. The requirement of cell-dependent forces to generate FN fibrils restricts investigation of the mechanism of assembly. To address this, we use a recently developed biophysical model of fibrillogenesis to test competing hypotheses for the location and number of cryptic FN-FN binding sites and quantify the effect of these molecular alterations on assembled FN fibril properties. Simulations predict that a single FN-FN binding site facilitates either negligible fibrillogenesis or produces FN fibrils that are neither robust nor physiological. However, inclusion of multiple FN-FN binding sites predicts robust fibrillogenesis, which minimally depends on individual domain properties. Multiple FN-FN binding site models predict a heterogeneous fibril population that contains two distinct phenotypes with unique viscoelastic properties, which we speculate may play a key role in generating heterogeneous mechanical signaling in the extracellular matrix of developing and regenerating tissues.

## Introduction

Soluble fibronectin (FN) is present in blood plasma at high concentration and is assembled by cells into insoluble, elastic fibrils, which play a major role in cell migration, cell adhesion, and formation of a provisional extracellular matrix (reviewed in the literature)^1–4^. FN fibril assembly serves as an early and crucial step in embryogenesis^5–7^ and wound healing^8,9^, while misregulation of FN assembly is associated with diseases, including cancer^10^, liver disease^11^, and lung disease^12^. Assembly of FN fibrils requires the application of cell-generated traction forces via integrins^13^: cells bind to FN via transmembrane integrins, which are coupled to the actin cytoskeleton via focal adhesions. Myosin-driven contractility applies force to the focal adhesion complex, which leads to deformation of the attached FN dimer, identified as essential for FN fibrillogenesis^13,14^.

**Figure 1:**
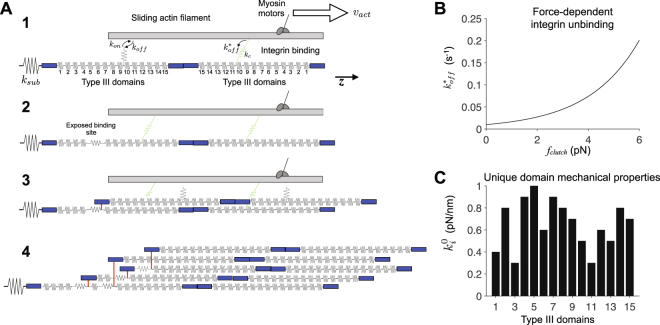
Diagram and key properties of the fibronectin assembly model. (**A**) Illustration of fibril assembly. 1. Assembly begins with a single fibronectin (FN) molecule, represented by 30 springs in series, attached to an elastic substrate, with stiffness *k*_*sub*_. Myosin motors pull on the actin filament at velocity *v*_*act*_ along the z-axis. Integrins (i.e., molecular clutches) reversibly bind the actin filament with rates *k*_*on*_ and *k*_*off*_. Bound integrins transmit a force proportional to the clutch stiffness *k*_*c*_, and unbind with a force-dependent off-rate ${k}_{off}^{\ast }$. Note that integrin springs are connected in parallel with springs representing FN Type III domains. 2. Actomyosin-driven stretch FN Type III domains, exposing a cryptic FN binding site. 3. A soluble FN molecule in the extracellular space binds to the exposed binding site. 4. Subsequent integrin binding, FN Type III domain stretching, and FN-FN binding events produce an elastic, insoluble FN fibril. Adapted from Weinberg *et al*.^40^. (**B**) In contrast with the constant integrin binding on-rate *k*_*on*_, integrin unbinding off-rate ${k}_{off}^{\ast }$ increases exponentially as a function of integrin spring or “clutch” force *f*_*clutch*_, given by ${k}_{off}^{\ast }={k}_{off}\exp ({f}_{clutch}/{f}_{b})$, where *k*_*off*_ is the integrin unbinding rate in the absence of force and *f*_*b*_ is a characteristic “break” force. (**C**) Each Type III domain has a unique characteristic resting stiffness ${k}_{i}^{0}$, for *i* = 1, 2, …, 15.

Earlier studies demonstrating that FN fibrils only assemble when FN molecules are subjected to cell contractile forces suggests that there is a buried cryptic binding site in FN molecules that is only exposed when under tension^13,14^. Once stretched, a cell-attached FN dimer can bind a soluble FN dimer. An FN dimer is comprised of two disulfide bonded FN monomers, which each consist of a series of individually folded domains that comprise one of three structures, which are referred to as Type I, II, or III (Fig. 1A1). The soluble FN dimer binds via the 70 kDa amino-terminal region of FN, which is comprised of Type I and Type II domains, to an exposed cryptic binding site in the growing FN fibril^15–18^. The FN binding site in the **fibrillar** FN is thought to be contained within the 15 Type III domains of FN. However, several Type III domains have been implicated in this role: studies have suggested that the critical binding site is in either III_1_ or III_2_^19–23^; however, deletion of III_1–7_ still results in FN fibril assembly^21^, albeit at a reduced rate. Other studies have demonstrated binding between the 70 kDa fragment of FN and III_10_^19^, III_12–14_^24^, and III_4–5_^25^. This suggests that multiple Type III domains may be capable of binding to soluble FN and facilitating fibrillogenesis.

The 70 kDa N-terminus of FN is capable of binding to various proteins via *β*-strand addition^26–31^. Steered molecular dynamics (SMD) simulations have indicated that stretched FN Type III domains have a stable intermediate conformation in which *β*-strands along the edges of the domain are extended and exposed^32,33^. This conformation would be capable of binding other proteins by *β*-strand addition. Since *β*-strand addition consists exclusively of backbone hydrogen bonding, it is reasonable to envision a mechanism by which all 15 Type III domains are capable of binding the 70 kDa N-terminus.

While all 15 Type III domains in FN have a homologous structure, biophysical studies have demonstrated that the domains vary widely in their mechanical and chemical stability^34–36^. Given the similar structure of all Type III domains, it is feasible to hypothesize that all 15 domains may be capable of FN-FN binding, and that the degree of binding would be determined by the relative magnitudes of the domain stiffness. Work from Harold Erickson’s group has demonstrated that several FN Type III domains are opened during FN fibrillogenesis^37^, while work from the Ingber group work has indicated a specific role of the ‘B’ *β*-strand in facilitating FN fibrillogenesis^38,39^. Taken together, there is a strong rationale that FN fibrillogenesis may be facilitated by stretching of FN Type III domains, exposure of *β*-strands, and subsequent *β*-strand addition of the 70-kDa N-terminus of a soluble FN molecule.

Our group has recently developed a computational model of FN assembly that predicts FN fibril growth from first principles of applied traction force and domain unfolding^40^. Briefly, the model simulates FN fibril growth by modeling each FN dimer as a series of Hookean springs that represent each elastic Type III domain (Fig. 1A1). Integrins bind to the III_10_ domain via a first-order reversible reaction that has a force-dependent off-rate (Fig. 1A1,B). Integrins are stretched via strains applied from simulated actomyosin forces, which follow an inverse force-velocity relationship. Strains result in deformation of the FN dimer, which deform the Type III domains, each of which has a unique domain stiffness ${k}_{i}^{0}$ (Fig. 1C). Following domain stretch beyond a cryptic binding site exposure threshold length *ε*_*t*_ (Fig. 1A2), a domain can bind a soluble FN dimer via a first-order stochastic reaction (Fig. 1A3). As fibril growth progresses (Fig. 1A4), the collective fibril traction force serves as a feedback on the actomyosin force. FN fibrils bind in a hexagonal packing array, and as the fibril grows, integrin binding and soluble FN dimer binding are limited the the “perimeter” of the fibril. Additional details of the model and simulations are provided in Methods below.

**Figure 2:**
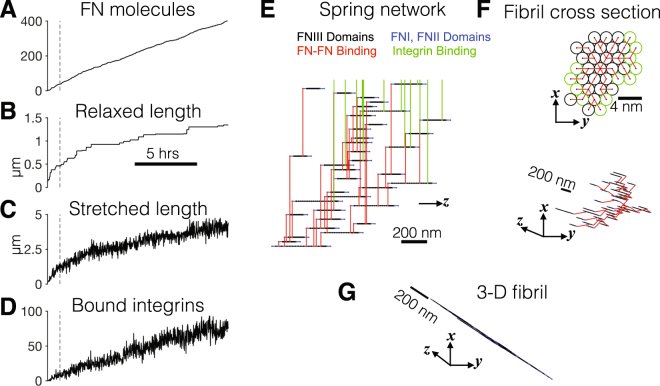
Morphometrical and biomechanical structure and properties during fibronectin (FN) fibril assembly. (**A**) The number of FN molecules, (**B**) relaxed length, (**C**) stretched length, and (**D**) number of bound integrins are shown as a function of time for a representative simulation of an assembling FN fibril with cryptic binding sites in all 15 Type III domains. (**E**) The Hookean spring network connections along the z-axis are shown: elastic FN type III domains (black), FN-FN binding (red), inelastic FN type I and II domains (blue), and integrin binding (green) are shown, after one hour of simulation time (vertical dashed line in **A**–**D**). (**E**) (top) The FN fibril cross-section in the x-y plane is shown, with FN-FN connections (red). “Perimeter” FN molecules with bound integrins are shown in green, while all other FN molecules are shown in black. (bottom) The FN fibril cross-section is expanded in x-y-z space. (**G**) The three-dimensional FN fibril architecture is shown.

This model provides a platform to investigate how the location of FN-FN binding sites, the number of FN-FN binding sites, and the mechanical properties of Type III domain unfolding affect the morphology and mechanics of assembled FN fibrils. In this study, we use this model to systematically probe how the number of Type III domain cryptic binding sites and their respective mechanical and chemical binding properties regulate FN fibril assembly. A representative simulation example with cryptic binding sites present in all 15 Type III domains is shown in Fig. 2. Time-series measurements show that the number of FN molecules comprising the fibril increases approximately linearly in time (Fig. 2A). The fibril relaxed length (*L*_*r*_, the total fibril length in the absence of actomyosin mediated forces) and the stretched length (*L*_*s*_, the total length in the presence of these forces) increase in time, approaching equilibrium values after several hours (Fig. 2B,C). The relaxed length strictly increases in time and is calculated directly from the Hookean spring network architecture, while the stretched length fluctuates in time due to frequent and stochastic integrin binding/unbinding events (Fig. 2D). The total number of bound integrins tends to increase in time as more FN molecules, and thus more available integrin binding site sites on the fibril “perimeter,” comprise the fibril. The fibril spring network is shown after one hour in Fig. 2E, with individual Type III domains, FN-FN binding, and integrin binding shown with black, red, and green lines, respectively, while the inelastic Type I and II domains are shown in blue. The fibril cross section shows the relative location of individual FN molecules, with FN molecules with bound integrins shown in green (Fig. 2F), and the 3D fibril illustrates the size of the assembling fibril (Fig. 2G).

## Results

### Single FN-FN binding site models do not predict robust fibrillogenesis

**Figure 3:**
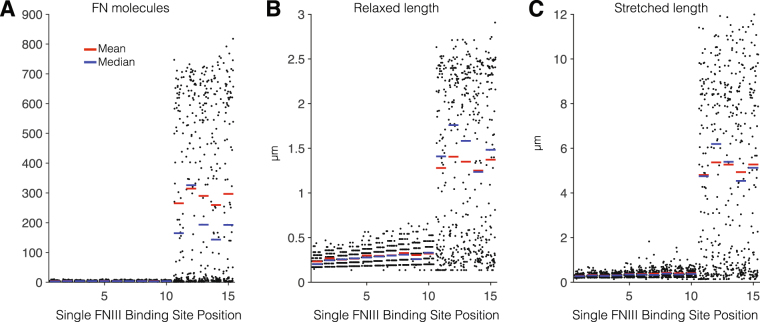
Fibronectin (FN) morphometry of fibrils with a single cryptic binding site. (**A**) The number of FN molecules, (**B**) relaxed length, and (**C**) stretched length for a single cryptic binding site located in domain III_1_ through III_15_. Negligible fibrillogeneis occurs in fibrils with a single binding site N-terminal of or at III_10_ (the integrin binding position). For fibrils with a single binding site C-terminal of III_10_, fibrillogenesis occurs and produces both small and large fibrils but also fails in approximately 40% of simulations (see Fig. 5). Each black dot represents results from one simulation (100 simulations, for each Type III domain binding site).

To test the contribution of different cryptic FN-FN binding sites, we first simulated fibrils in which only a single FN-FN binding site was present. We systematically consider fibril assembly with a single binding site present in each of the 15 Type III domains. Simulation measurements show the number of FN molecules in each fibril, the relaxed fibril length, and the stretched fibril length (Fig. 3). We observe two very distinct regimes: fibrils with the single Type III domain binding site N-terminal of or at the III_10_ domain and fibrils with the binding site C-terminal of the III_10_ domain, which is the critical domain at which the FN-integrin binding occurs. Thus, the position of the FN-FN binding site, relative to the FN-integrin binding site, is a critical factor in FN assembly. For fibrils with the binding site N-terminal of or at the III_10_ domain, we find negligible assembly. All fibrils contain less than 10 FN dimers and have relaxed lengths less than 1 *μ*m. For fibrils with the binding site C-terminal of the III_10_ domain, we find three responses: assembly of a small fibril, assembly of a larger fibril, and negligible fibril assembly. These results collectively suggest that a cryptic FN-FN binding site located in at least one Type III domain C-terminal of the integrin binding site, and thus positioned between the two integrin binding sites in the FN dimer (see Fig. 1A1), is critical and necessary for fibrillogensis.

**Figure 4:**
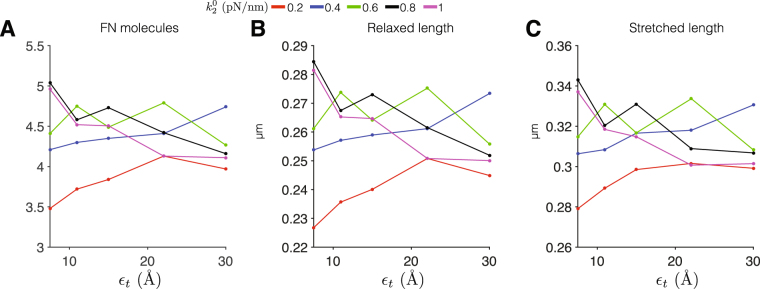
Altered Type III domain properties do not promote fibrillogenesis in fibronectin with a single binding site N-terminal of III_10_. Mean values from 100 simulations for (**A**) the number of fibronectin (FN) molecules, (**B**) relaxed length, and (**C**) stetched length are shown for varying FN-FN binding site exposure threshold *ε*_*t*_, for different values of resting domain stiffness ${k}_{2}^{0}$ for fibronectin with a single cryptic binding site in III_2_. Baseline parameter values: ${k}_{2}^{0}=0.8$ pN/nm, *ε*_*t*_ = 15 Å.

We investigate this point further below. However, first, we address an important question: does our observation of negligible assembly occurring for a single binding site N-terminal of III_10_ depend on the mechanical and chemical binding properties of that Type III domain? In other words, are these results a consequence of two model parameters: 1) the mechanical stiffness of the domain, and 2) the domain stretch length required to exposes the cryptic FN-FN binding site? To address this question, we vary the values of resting domain stiffness ${k}_{i}^{0}$ and FN-FN binding site exposure threshold *ε*_*t*_, specifically considering the III_2_ domain. Note that we expect similar results for any of the individual Type III domains N-terminal of III_10_. In Fig. 4, we plot the averages for the number of FN molecules and relaxed and stretched length, varying resting domain stiffness of the III_2_ domain (${k}_{2}^{0}$) from 0.2 to 1 pN/nm (the full range of resting domain stiffness values) and scaling *ε*_*t*_ by a factor of 2 above and below the nominal threshold of 15 Å. While there are some general trends observed, the primary result is that, regardless of the values for ${k}_{2}^{0}$ and *ε*_*t*_, FN assembly was negligible, further suggesting that a single binding site N-terminal of III_10_, regardless of the mechanical or chemical binding properties, cannot result in significant FN fibrillogenesis.

**Figure 5:**
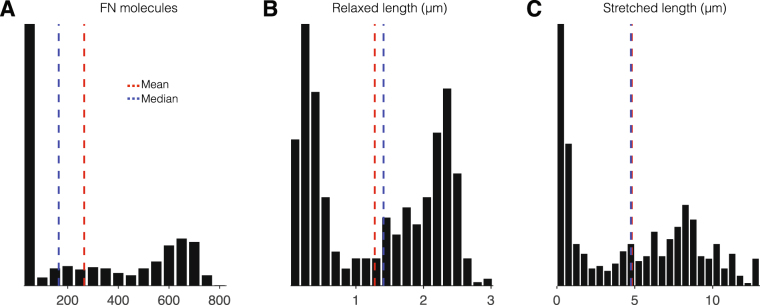
Non-robust fibrillogenesis in FN with a single binding site in III_11_. Histograms from 500 simulations for (**A**) the number of FN molecules, (**B**) relaxed length, and (**C**) stretched length length illustrate the large fraction of simulations that yield negligible assembly and two sub-populations of small and large FN fibrils. Similar results were observed for III_12_, III_13_, III_14_, and III_15_ (data not shown).

We next investigate in more detail the properties of FN assembly for a single binding site C-terminal of III_10_. We plot histograms for the number of FN molecules and relaxed and stretched length for fibrils with a single binding site at the III_11_ domain (Fig. 5). The FN molecules histogram illustrates the three sub-populations: one large peak representing no or negligible assembly and two smaller sub-populations, one with smaller fibrils (peak at 200 molecules) and one with larger fibrils (peak at 650 molecules). The distributions are less distinguishable when considering the resting and stretched lengths, but two distinct groups (negligible assembly/small fibrils and large fibrils) are still discernible. However, the population of fibrils in which no or negligible assembly occurs is, in fact, the largest group: 43% of fibrils have less than 25 FN molecules and 45% are less than 1 *μ*m. Similar values are found for single binding sites located in III_12_, III_13_, III_14_, and III_15_ (not shown). These results suggest that, while a single binding site C-terminal of III_10_ can result in fibrillogenesis, the FN fibril assembly process is fragile, failing nearly half of the time. Physiologically, FN assembly is a robust and reproducible process, suggesting alternative hypotheses for the location of FN-FN binding sites: multiple cryptic FN-FN binding sites are located within the 15 Type III domains.

### Multiple FN-FN binding site models predict robust fibril assembly with distinct sub-populations

While testing all possible combinatorial combinations of multiple binding sites is not feasible (2^15^–16 = 32752 combinations), we consider several combinations of previously identified sites, along with our hypothesis of a binding site located in all 15 Type III domains. Specifically, we consider the following four (4) hypotheses for FN-FN binding: (1) binding sites located in each of the first three Type III domains (III_1–3_); (2) binding sites located in all of the Type III domains N-terminal of III_10_ (III_1–9_); (3) binding sites located in the first three Type III domains and one domain C-terminal of III_10_ (III_1–3,11_), and (4) binding sites located in all 15 Type III domains (III_1–15_). For each hypothesis, we characterize the fibril size and morphometry; extensibility, given by the stretched-to-relaxed length ratio (*L*_*s*_/*L*_*r*_); and geometry, characterized by the relaxed length-to-thickness (*L*_*r*_/*T*) ratio. Average values for fibrils assembled with these subsets of multiple FN-FN binding sites are shown in Table 1, with two single binding cases shown for comparison.

**Table 1:** Mean ± standard error values for fibril size, morphometry, and geometry with single or multiple cryptic FN-FN binding sites. Statistics are from 100 simulations (row 1), 500 simulations (for row 2–5), and 1000 simulations (for row 6).

FN-FN Binding Sites	FN molecules	Relaxed length (*μ*m)	Stretched length (*μ*m)	Extensibility	*L*_*r*_/*T* ratio
III_2_	4.6 ± 0.18	0.27 ± 0.007	0.33 ± 0.010	1.21 ± 0.001	50.4 ± 0.52
III_11_	265 ± 12.5	1.28 ± 0.040	4.81 ± 0.18	3.69 ± 0.25	50.4 ± 0.24
III_1–3_	336 ± 1.2	0.94 ± 0.004	1.68 ± 0.009	1.79 ± 0.006	22.4 ± 0.11
III_1–9_	376 ± 2.9	1.13 ± 0.006	3.05 ± 0.027	2.68 ± 0.015	25.7 ± 0.092
III_1–3,11_	380 ± 3.9	1.24 ± 0.007	3.62 ± 0.033	2.90 ± 0.016	28.3 ± 0.13
III_1–15_	416 ± 3.8	1.39 ± 0.007	4.53 ± 0.035	3.23 ± 0.013	30.5 ± 0.090

**Figure 6:**
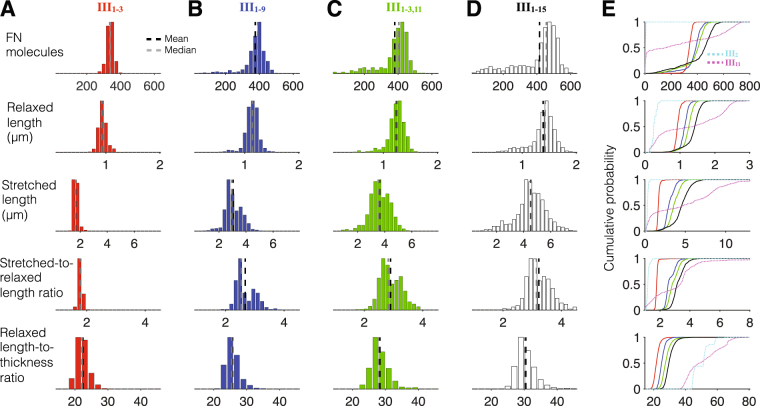
Fibronectin with multiple Type III domain binding sites yield robust fibrillogenesis. Histograms for (row 1) the number FN molecules, (row 2) relaxed length, (row 3) stretched length, (row 4) stretched-to-relaxed length ratio, and (row 5) relaxed length-to-thickness ratio are shown for fibronectin with binding sites in (**A**, solid red in **E**) III_1–3_; (**B**, solid blue) III_1–9_; (**C**, solid green) III_1–3,11_; and (**D**, solid black) III_1–15_. Cumulative probability distributions are shown in (**E**), with distributions for fibronectin with single binding sites in III_2_ (dashed cyan) and III_11_ (dashed magenta) shown for comparison. Histograms are from 500 simulations (for **A**–**C**) and 1000 simulations (for **D**).

For fibrils with binding sites in III_1–3_, we observe robust assembly for a fairly homogeneous population of small and “squat” fibrils (Fig. 6A, red), with a distribution peak at 350 FN molecules. Thus, while our model does predict robust fibrillogenesis for the fibrils with these three binding sites, the fibrils are smaller and less extensible compared with experimental measures and observations^40,41^. We next consider fibrils with additional cryptic binding sites (III_1–9_, Fig. 6B, blue). These additional binding sites, on average, increase the fibril size, with the peak distribution shifted to 400 FN molecules. On average, the fibrils are more extensible and “skinnier,” compared with the three binding site model. Additionally, the FN molecule distribution demonstrates a more prominent left tail, indicating assembly of more smaller fibrils; however this sub-population of small fibrils is a relatively small percentage of all fibrils assembled.

For fibrils with three N-terminal and one C-terminal Type III domains (III_1–3,11_, Fig. 6C, green), we find an increase in the size of large extensible fibrils, as the FN molecule distribution peak shifts to 425 molecules. Further, we observe a more distinct sub-population of smaller fibrils, with a peak near 200 FN molecules. Thus, the combination of multiple cryptic binding sites N-terminal and one C-terminal of III_10_ produces a heterogeneous fibril population with both small and large fibrils.

Finally, we consider the hypothesis of cryptic binding sites located in all 15 Type III domains (III_1–15_, Fig. 6D, black), and we observe the same general trends: the large fibrils, on average, are larger, with the FN molecule peak distribution shifted to 475 molecules. Further, the small fibril sub-population is more prominent as well. Thus, the presence of cryptic binding sites in all 15 Type III domains results in robust fibrillogenesis, producing a heterogeneous population, with extensible large and small fibrils. We plot the cumulative distributions for each measure in Fig. 6E, highlighting the heterogeneous fibril population for all 15 binding sites (black), with the distributions for fibronectin with a single III_2_ or III_11_ binding site shown for comparison (dashed cyan or magenta, respectively).

Thus, our results strongly suggest that multiple FN binding sites are required for robust fibrillogenesis. Further, the presence of at least one binding site C-terminal of the integrin binding site in III_10_ is critical for generation of a heterogeneous population of FN fibrils. The presence of cryptic binding sites located in all Type III domains results in two sub-populations of small and large fibrils. Our analysis in Fig. 4 demonstrated that varying the properties of Type III domains with a single cryptic binding site did not greatly influence fibrillogenesis or fibril properties (Fig. 4); regardless of mechanical and chemical binding properties, negligible assembly occurred. We next investigated whether these properties affect the assembled fibril population when multiple binding sites are involved, and also investigated whether the relative magnitudes of mechanical properties in neighboring domains is important for FN fibrillogenesis. In other words, each Type III domain has a unique mechanical stiffness, and the domains are arranged in a particular order; is the order and variation in mechanical stiffness important in FN fibrillogenesis?

### Fibril assembly is minimally affected by the relative stiffnesses of Type III domains and domain order

We consider six (6) variations on fibrils with binding sites in all 15 Type III domains: (1) domain stiffnesses are scrambled: the same 15 domain stiffness values are used, but for each simulation, the specific order has been randomly rearranged; (2) domain stiffnesses for all 15 Type III domains are set to a constant, equal to the *minimum* value of the 15 stiffness values in the baseline case; (3) domain stiffness for all 15 Type III domains are set to the *mean* value from the baseline case; (4) domain stiffness for all 15 Type III domains are set to the *maximum* value from the baseline case; (5) baseline domain stiffness values are maintained, but the FN-FN binding site exposure threshold *ε*_*t*_ is half of the nominal threshold of 15 Å; or (6) baseline domain stiffness values are maintained, but the FN-FN binding site exposure threshold *ε*_*t*_ is twice the nominal threshold of 15 Å. Average values for fibril size, morphometry, and geometry for these variations on the Type III domain properties are shown in Table 2.

**Table 2:** Mean ± standard error values for fibril size, morphometry, and geometry baseline or altered Type III domain mechanical and chemical properties. Statistics are from 500 simulations (for row 3, 5, 6, 7) and 1000 simulations (for row 1, 2, 4).

Fibril Type III Domains	FN molecules	Relaxed length (*μ*m)	Stretched length (*μ*m)	Extensibility	*L*_*r*_/*T* ratio
Baseline	416 ± 3.8	1.39 ± 0.0070	4.53 ± 0.035	3.23 ± 0.01	30.5 ± 0.090
Scrambled	418 ± 3.7	1.39 ± 0.0067	4.57 ± 0.035	3.24 ± 0.01	30.6 ± 0.089
Constant ${k}_{i}^{0}={\kappa }_{min}$	403 ± 6.0	1.37 ± 0.011	4.48 ± 0.054	3.23 ± 0.02	30.8 ± 0.13
Constant ${k}_{i}^{0}={\kappa }_{mean}$	402 ± 4.0	1.36 ± 0.0072	4.41 ± 0.035	3.20 ± 0.01	30.6 ± 0.10
Constant ${k}_{i}^{0}={\kappa }_{max}$	401 ± 5.4	1.36 ± 0.0095	4.41 ± 0.048	3.21 ± 0.02	30.5 ± 0.12
Reduced *ε*_*t*_ = 7.5 Å	424 ± 5.2	1.44 ± 0.010	4.83 ± 0.051	3.31 ± 0.02	31.3 ± 0.13
Increased *ε*_*t*_ = 30 Å	411 ± 5.2	1.34 ± 0.0092	4.31 ± 0.046	3.19 ± 0.02	29.6 ± 0.13

**Figure 7:**
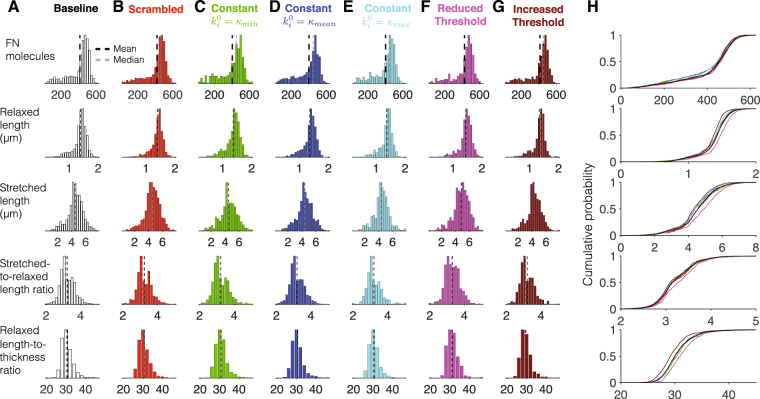
Fibronectin with 15 Type III binding sites minimally depends on domain mechanical and chemical properties. Histograms for (row 1) the number FN molecules, (row 2) relaxed length, (row 3) stretched length, (row 4) stretched-to-relaxed length ratio, and (row 5) relaxed length-to-thickness ratio are shown for fibronectin with (**A**) baseline properties; (**B**) scrambled domain order; constant domain resting stiffness (**C**) ${k}_{i}^{0}={\kappa }_{min}$, (**D**) ${k}_{i}^{0}={\kappa }_{mean}$, and (**E**) ${k}_{i}^{0}={\kappa }_{max}$; FN-FN binding site exposure threshold *ε*_*t*_ (**F**) reduced to 7.5 Å and (**G**) increased to 30 Å. Cumulative probability distributions are shown in (H). Colors correspond with those shown in **A**–**G**. Histograms are from 500 simulations (for **C**,**E**,**F**,**G**) and 1000 simulations (for **A**,**B**,**D**).

We plot histograms and the cumulative distributions for key morphometrical measurements for fibrils assembled with baseline domain order and properties (i.e., Fig. 6D, replotted in Fig. 7A for comparison) and the six variations. The histogram and cumulative distributions illustrate small differences in fibrils assembled with FN with varying domain properties. For example, scrambling domain stiffness values (Fig. 7B) or reducing FN-FN binding site exposure threshold *ε*_*t*_ (Fig. 7F) slightly increases average fibril size, compared with baseline fibrils. In contrast, constant domain stiffnesses (Fig. 7C–E) or increasing *ε*_*t*_ (Fig. 7G) slightly decreases average fibril size. We also find small changes in fibril extensibility and geometry. However, more interestingly, these differences in fibril size and properties are small. Histograms illustrate that populations for all variations considered are fairly similar, with a large peak representing a sub-population of large fibrils and a smaller, broader peak representing a sub-population of smaller fibrils, with similar overall fibril extensibility and geometry. Thus, our simulations suggest that for fibrils with FN binding sites in all 15 Type III domains, while the specific mechanical and chemical binding domain properties do slightly influence overall fibril population characteristics, the fibrillogenesis process is robust, producing a heterogeneous fibril population, independent of individual domain properties.

### Viscoelastic properties of FN fibrils

Our predictions pose a critical question: What is the biological significance of this heterogeneous fibril population? To answer this question, we further investigate the properties of the simulated population of assembled FN fibrils. While it has long been appreciated that the elastic modulus of tissue facilitates cell signaling^42–45^, recent studies have suggested that the non-linear components of ECM may play an equally important role^46–48^. These recent studies have demonstrated the viscoelastic behavior of ECM gels^46,47^; however to our knowledge, no work has specifically investigated the viscoelastic properties of individual fibrils. To examine these FN fibril viscoelastic properties, we conducted an additional set of simulations. Fully assembled fibrils were relaxed in the model by removing all integrin attachments; then fibrils were “re-stretched” by reactivating cell attachment. This models the situation where an assembled fibril remains after a cell has migrated from the spot of assembly and predicts the effects of the fibril being re-stretched by another cell.

**Figure 8:**
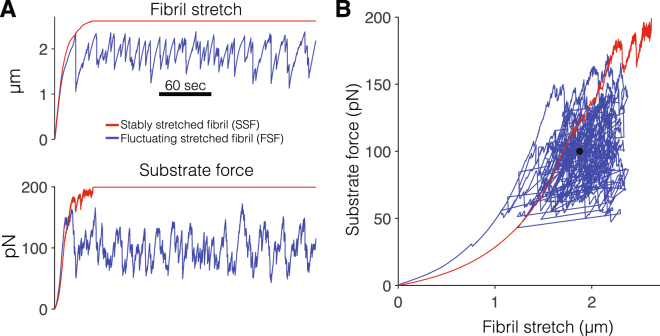
Two sub-types of viscoelastic fibronectin fibrils. (**A**) Fibril stretch (difference of the stretched and relaxed length) and substrate force are shown as a function of time for two fibril sub-types observed in simulations: stably stretched fibrils (SSFs, red) and fluctuating stretched fibrils (FSFs, blue). See text for details. (**B**) Substrate force is shown as a function of fibril stretch for the representative SSF and FSF. The solid black circle represents the FSF mean substrate force and fibril stretch during the final two minutes of simulation time.

Interestingly, in response to re-stretching from rest, we observe two distinct fibril sub-types; representative examples are shown in Fig. 8. In the fibril which we termed a *stably stretched fibril* (SSF), the fibril stretch (the difference between the stretched and relaxed length, Δ*L* = *L*_*s*_ − *L*_*r*_) gradually increases as a function of time, before approaching a constant or stable value, while the substrate traction force (*f*_*sub*_ = *k*_*sub*_ *x*_*sub*_, the product of the substrate stiffness *k*_*sub*_ and substrate stretch *x*_*sub*_) also gradually increases, with a few deflections, before reaching the stall force (here, 200 pN) that prevents further actomyosin-mediated fibril stretch (Fig. 8A, red). In contrast, in the fibril which we termed a *fluctuating stretched fibril* (FSF), both fibril stretch and substrate force initially gradually increase, followed by persistent fluctuation around an “average” stretch and substrate force value (Fig. 8A, blue). These fluctuations occur due to the stochastic formation and breaking of bonds between integrins and the FN fibril. The viscoelastic properties of these fibrils can be represented by the substrate force plotted against fibril stretch (Fig. 8B). We also denote the average FSF stretch and substrate force value during fluctuation (black circle). Both fibril types (FSFs and SSFs) demonstrate non-linear stiffness. Interestingly, the resting stiffness (i.e., the slope of the force-stretch curve at stretch of 0) is larger for the FSF, despite maintaining an average substrate force less than the SSF.

**Figure 9:**
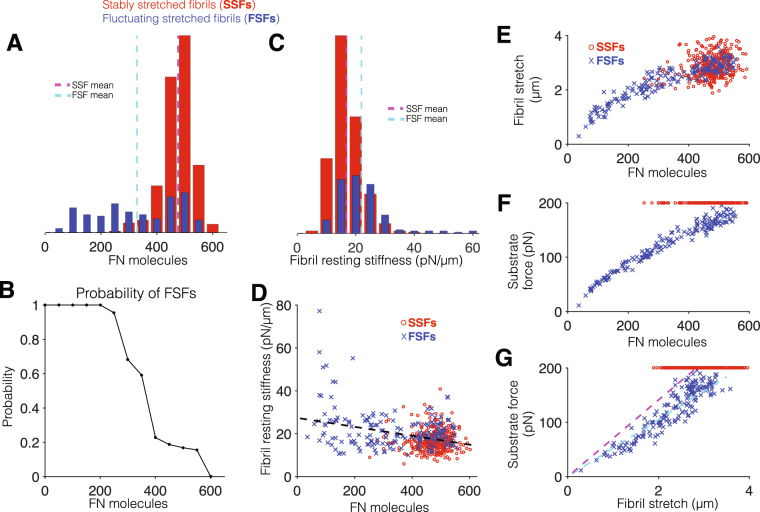
Viscoelastic properties of assembled fibronectin fibrils. (**A**) Histogram of the number of fibronectin (FN) molecules, for stably stretched fibrils (SSFs, 362 fibrils) and fluctuating stretched fibrils (FSFs, 169 fibrils). (**B**) Probability of an FSF, as a function of the number of FN molecules. (**C**) Histogram for SSF and FSF resting stiffness values (see text for details). (**D**) SSF (open red circles) and FSF (blue x’s) resting stiffness values are negatively correlated with the number of FN molecules. (**E**) Fibril stretch (difference of stretched and resting length) and (**F**) substrate force are shown as a function of the number of FN molecules, for SSFs (red) and FSFs (blue). (**G**) Substrate force shown as a function of fibril stretch for SSFs (red) and FSFs (blue). Lines with the slope of best fit for SSFs and FSFs are shown in dashed magenta and cyan, respectively.

The properties of SSFs and FSFs are summarized in Fig. 9. SSFs comprised the majority of fibrils (68%). Histograms of the number of FN molecules in SSFs and FSFs illustrates two fibril sub-populations (Fig. 9A). In general, SSFs are larger fibrils, comprised of more FN molecules (476 ± 2.8 molecules, vertical dashed magenta), compared with the smaller FSFs (329 ± 11.5 molecules, vertical dashed cyan). Thus, the larger the fibril, i.e., more FN molecules, the less likely the fibril is an FSF and more likely it is an SSF (Fig. 9B). Consistent with Fig. 8B, the fibril resting stiffness for FSFs is typically larger (mean of 22.0 ± 0.71 pN/*μ*m), compared with SSFs (mean of 16.8 ± 0.25 pN/*μ*m) (Fig. 9C). Further, amongst all fibrils, we find a negative correlation between the fibril resting stiffness and the number of FN molecules (Pearson correlation coefficient *r* = −0.349, Fig. 9D), indicating that smaller fibrils tend to have a higher resting stiffness.

We plot the FSF average fibril stretch (Fig. 9E, blue x’s) and substrate force (Fig. 9F, blue x’s) during fluctuation (denoted by the black circle in Fig. 8B) as a function of the number of FN molecules in the fibril and find a close positive correlation for both (*r* = 0.924 and 0.980, respectively). In contrast, there is a much weaker correlation between the number of FN molecules in SSFs and the stable fibril stretch (Fig. 9E, open red circles, *r* = 0.217) and no correlation with the stable substrate force (equivalent to the stall force of 200 pN for all fibrils). The substrate force and fibril stretch for the FSFs are also positively correlated (*r* = 0.922), with a slope of 52.5 ± 0.63 pN/*μ*m (Fig. 9G). The slope of the substrate force-fibril stretch relationship provides a measure of the average FSF stiffness during fibril fluctuation. The average SSF slope (based on the stable fibril stretch and substrate force) is equal to 70.6 ± 0.55 pN/*μ*m. Thus, interestingly, the resting stiffness of FSFs is more rigid compared with the SSFs (Fig. 9C), while the average FSF stiffness is softer (Fig. 9G), illustrating complex viscoelastic properties in the two fibril sub-types.

## Discussion

Despite decades of research, the mechanism of FN assembly is not well understood. While it has been established that FN fibrils require application of cell-derived traction force^13^, and that these appear to expose buried cryptic binding sites within the Type III domains of FN, there have been conflicting results regarding the number and location of these binding sites. Here, we have used our recently developed model of FN fibrillogenesis^40^ to investigate how the number and location of FN-FN binding sites alters the predicted assembly of FN fibrils.

Our results demonstrate that a single binding site located N-terminal of or at III_10_ facilitates negligible FN fibrillogenesis. In contrast, a single binding site located C-terminal of III_10_ is capable of generating fibrils; however, these fibrils are small, are only seen in a small percentage of simulations, and are not comparable to those seen physiologically. These findings provide a mechanistic explanation of previous experimental studies that suggest that multiple Type III domains contribute to robust fibronectin fibrillogenesis^20,21^. Our model also provides mechanistic support to the theorized necessity of an FN-FN binding site located C-terminal of III_10_, which was hypothesized nearly 20 years ago in one of the original works that demonstrated the need for contractile force in FN fibrillogenesis^13^. Our model predicts that these results are not significantly affected by altering the stiffness of the Type III domain or the stretch required for FN-FN binding.

Further results demonstrate that simulations containing multiple FN-FN binding sites produce much more robust, physiologically representative fibrils. Models that contained multiple binding sites and had at least one binding site C-terminal of III_10_ generated fibrils that best matched experimentally observed extensibility of four-fold stretch^20^. Interestingly, these results were not significantly affected by the order of the domain stiffness values within the FN molecule: models in which the stiffness values were randomly reordered did not show significant differences in resulting fibrils. Similarly, results were not affected by variation in stiffness values between domains: when all 15 domains were set to a constant value, fibrillogenesis was similar to the baseline case. This suggests that robust FN fibrillogenesis requires multiple FN-FN binding sites, but neither the individual mechanical properties of each domain nor the order of those stiffness values plays a dramatic role in FN fibrillogenesis.

Taken together, these results provide novel insight into the mechanism of fibronectin fibrillogenesis, and support experimental evidence that suggests that multiple cryptic binding sites within FN fibrils are required for robust fibrillogenesis. This mechanistic insight addresses an extensively debated aspect of fibronectin biology, and could have significant implications in understanding how cell-derived forces direct fibronectin fibrillogenesis.

Models that exhibited robust FN fibrillogenesis also demonstrated a heterogeneous population of fibrils. What is the significance of a heterogeneous population of FN fibril sizes? We hypothesized that this diverse array of fibril sizes may also lead to diversity in the mechanical properties of individual FN fibrils. The mechanical properties of FN fibrils could have profound impacts in the field of mechanobiology, where the importance of non-linear elasticity and the subsequent effects on cellular function is only beginning to be understood. Recent studies have demonstrated the non-linearity of extracellular matrix fibrils, and have suggested that these mechanical properties may be crucial for unique signaling events in the healing wound or developing embryo^46,47^. Future studies from our group will probe how these unique mechanical properties affect cellular signaling and function.

Simulations in which assembled fibrils were “re-stretched” indicated two distinct fibril phenotypes: one which we have termed “stably stretched fibrils” or SSFs, which exhibit constant stretch and force, and one which we have termed “fluctuating stretched fibrils” or FSFs, which fluctuate with time in terms of stretch and force. Results show a correlation with size, with larger fibrils more likely to be SSFs. Interestingly, the two fibril phenotypes show distinct mechanical properties: both phenotypes exhibit non-linear stiffness, but FSFs exhibit larger stiffness values from rest compared to SSFs. This could potentially indicate a critical role for these smaller fibrils in situations of wound healing, fibrotic disease, and embryogenesis. Studies have indicated that cell differentiation into mesenchymal phenotypes, as is required during wound healing and embryogenesis, is enhanced on stiffer substrates^42,43^. As such, these small FSFs may represent an early event in presenting a stiffer mechanical cue to surrounding cells. Additionally, the unique non-linear elasticity of these two populations of fibrils could play a significant role in the mechanics of the provisional extracellular matrix, as previous studies have suggested^46,47^.

Our work suggests that FN fibrils require multiple FN-FN binding sites, are enhanced when at least one binding site is present at a site C-terminal of III_10_, and consist of a heterogeneous population of fibrils with varied mechanical properties. The current study will drive novel experimental efforts to identify FSFs and SSFs *in vitro* and *in vivo*, and quantify both their mechanical properties and subsequent cellular responses. We are currently developing experimental assays that can probe fibril mechanics to prove or disprove the existence of these two fibril subtypes. Given the significant role of FN fibrillogenesis in wound healing, embryogenesis, and fibrotic diseases, we envision that understanding these mechanical interactions between fibrils and cells may lead to novel insight into early stage events in each of these arenas.

## Methods

### Fibronectin Assembly Model Formulation

Simulations were performed using our recently developed model integrating FN assembly and cell-generated traction forces. Full details of model equations, parameters, implementation, and numerical methods can be found in Weinberg *et al*.^40^. We provide here an overview of the model. FN monomers are comprised of 29 independently folded domains, referred to as Type I, Type II, and Type III. While Type I and Type II domains are generally inelastic due to multiple disulfide bonds, the 15 Type III domains mediate the elastic properties of FN (Fig. 1A). Each Type III domain has unique resting mechanical properties and unfolds in response to force (Fig. 1C). FN molecules exist as a homodimer in the extracellular space, and thus in our model, we represent each individual FN dimer as 30 springs in series (one spring for each Type III domain, enumerated in sequence as III_1_ to III_15_, followed by III_15_ to III_1_). The Type III domain spring constants and forces are related by Hooke’s law and are time-dependent (as described in Weinberg *et al*.^40^).

Cells bind to individual FN molecules via transmembrane integrins, at a binding site in the III_10_ domain. Thus, each FN molecule contains two integrin binding sites, located at both III_10_ domains in the homodimer. Integrin binding is represented as a stochastic first-order reversible reaction, with constant on-rate and force-dependent off-rate (Fig. 1B). Integrin bonds are represented by a Hookean spring, termed a “molecular clutch” in prior models^40,49^. Upon binding, actomyosin forces stretch the bond spring, and via the III_10_ domain connection, stretch the assembling fibril. As the bond spring is stretched, the bond force increases, which exponentially increases the integrin binding off-rate and increasing the likelihood of a integrin bond rupture event. In this study, we investigate the cases in which a cryptic binding site is located in all 15 Type III domains, or specified subsets of the 15 Type III domains.

Simulations are initialized with a single FN molecule, at rest, bound to an elastic substrate with stiffness *k*_*sub*_ = 1000 pN/nm. As integrin-mediated binding stretches individual FN molecules, cryptic binding sites in specified Type III domains are exposed in a force-dependent manner (Fig. 1A2), enabling FN-FN binding and driving FN assembly (i.e., increasing the total number of FN molecules in the fibril). FN-FN binding is represented by a two-step process: 1) cryptic binding site exposure, represented as a stochastic process with probability predicted by a Hill-type equation with half-maximal response for domain threshold *ε*_*t*_; and 2) soluble FN binding to the exposed binding site, represented by a stochastic irreversible reaction. In a subset of simulations in this study, we increase or decrease the domain threshold *ε*_*t*_ in one or all Type III domains.

Prior experimental and computational studies suggest that the Type III domain stiffness changes as the domain unfolds^32–34^. Previous studies have demonstrated that Type III domains have unique mechanical unfolding properties; however these unique properties only exist until secondary structures are disrupted, after which point, domains behave as entropic springs that can be represented using the wormlike chain (WLC) model^50,51^. We account for both the domain-specific mechanical properties when domains are folded, and the domain-independent, WLC behavior at larger stretches, by specifying a stretch-dependent stiffness relationship in which the stiffness is a unique specific value at rest (${k}_{i}^{0}$ for *i* = 1,2, …, 15), and approaches an identical WLC-predicted stiffness as stretch increases (see Fig. 3 in Weinberg *et al*.^40^). In our prior work, we estimated the unique Type III domain resting stiffness values from several experimental studies^40^. In this study, we also investigate several variations for the Type III domain stiffness values, specifically 1) Type III domain stiffness value ${k}_{i}^{0}$ is varied; 2) all Type III domain stiffness values are non-unique (i.e., all equivalent); and 3) Type III domain stiffness values are reordered or scrambled from their baseline or control values.

### Computational Model Analysis

For each simulation, we quantify several important measures of fibril morphometry and mechanical properties, including the following: i) the number of FN molecules in the assembling fibril; ii) the relaxed or resting fibril length *L*_*r*_, which is determined from the Hookean spring network architecture assuming all springs are in their respective equilibrium position; iii) the stretched fibril length *L*_*s*_, the total fibril length under tension from actomyosin-induced forces; iv) the number of bound integrins; v) fibril thickness *T*, calculated from the fibril cross section diameter; and vi) substrate force *f*_*sub*_, given by the product of *k*_*sub*_ and substrate deflection *x*_*sub*_. We also calculate measures of extensibility *L*_*s*_/*L*_*r*_, given by the ratio of the stretched-to-relaxed length, and fibril geometry *L*_*r*_/*T*, given by the relaxed length-to-thickness ratio. Fibril measurements are averaged over the final 10 minutes preceding assembly termination, to account for stochasticity due to integrin bond formation and rupture. Mean values are presented plus/minus the standard error of the mean.

### Data availability

The datasets generated during and/or analyzed during the current study are available from the corresponding author on reasonable request.
